# Recent advances in understanding vertebrate segmentation

**DOI:** 10.12688/f1000research.12369.1

**Published:** 2018-01-23

**Authors:** Tomás Pais-de-Azevedo, Ramiro Magno, Isabel Duarte, Isabel Palmeirim

**Affiliations:** 1 Algarve Biomedical Center, Faro, Portugal; 2 CBMR, Centre for Biomedical Research, University of Algarve, Faro, Portugal; 3 Theoretical Biology and Bioinformatics, Utrecht University, Utrecht, Netherlands; 4 Department of Biomedical Sciences and Medicine, University of Algarve, Faro, Portugal

**Keywords:** Segmentation, Vertebrate, Somitogenesis, Clock, Wavefront, Embryo, Oscillation

## Abstract

Segmentation is the partitioning of the body axis into a series of repeating units or segments. This widespread body plan is found in annelids, arthropods, and chordates, showing it to be a successful developmental strategy for growing and generating diverse morphology and anatomy. Segmentation has been extensively studied over the years. Forty years ago, Cooke and Zeeman published the Clock and Wavefront model, creating a theoretical framework of how developing cells could acquire and keep temporal and spatial information in order to generate a segmented pattern. Twenty years later, in 1997, Palmeirim and co-workers found the first clock gene whose oscillatory expression pattern fitted within Cooke and Zeeman’s model. Currently, in 2017, new experimental techniques, such as new *ex vivo* experimental models, real-time imaging of gene expression, live single cell tracking, and simplified transgenics approaches, are revealing some of the fine details of the molecular processes underlying the inner workings of the segmentation mechanisms, bringing new insights into this fundamental process. Here we review and discuss new emerging views that further our understanding of the vertebrate segmentation clock, with a particular emphasis on recent publications that challenge and/or complement the currently accepted Clock and Wavefront model.

## Introduction

Development is the process by which multicellular organisms start out as just one cell, multiply into millions of others that biochemically communicate with each other, rearrange their positioning, and even die in a programmed way. Embryo segmentation represents a particularly interesting phase of development, where a collection of physical, chemical, and biochemical events are successfully orchestrated in space and time in order to develop a fully grown organism.

Segmentation is the partitioning of the body axis into a series of repeating units or segments. In vertebrates, this process occurs in the embryo by subdivision of the presomitic mesoderm (PSM) into metameric structures termed somites, although other segmented systems exist in vertebrates (for an excellent review on this subject, see Graham *et al*. ^1^). Segments in vertebrate embryos were first documented by the Italian biologist Marcello Malpighi in the 17th century ^2^, but, according to Verbout ^3^, it was not until the work of Francis Balfour in the late 19th century that the term “somite” (mesoblastic somites in the original writing) was first used to describe them ^4^. Somites form sequentially from rostral to caudal along the anterior-posterior (A-P) axis of the embryo, budding off in bilateral pairs from the unsegmented paraxial mesoderm on either side of the neural tube. These simple structures contain the precursor cells of the axial skeleton, musculature, connective tissue, blood vessel endothelium, and dermis of the vertebrate trunk, as well as muscles of the limbs ^5^. Each segment will give rise to a somite-base unit, each connected to the brain via individual nerve structures, allowing an organized projection of the A-P adult body in the brain, which is crucial for an optimized central/peripheral communication in the organism.

In this article, we review some of the recent progress made on the study of segmentation. We start by providing an overview of the evolution of segmentation in the animal kingdom, briefly discussing the current hypotheses for its origin. Then we discuss the mechanism of segmentation. We briefly introduce the 40-year-old model of somitogenesis—the Clock and Wavefront model—and discuss how recent studies have been reshaping its original formulation. We finish by providing a list of open questions. The brief nature of this review precludes it from being comprehensive, since not all important developments could be discussed in such a short format. Accordingly, we sincerely apologize to all authors whose relevant work is not cited for space considerations.

## Origin and evolution of segmentation

Overt body segmentation is considered a main evolutionary innovation occurring in three major animal phyla—annelids, arthropods, and chordates (reviewed in 6). By the end of the 19th century, these classical segmentation processes were viewed as homologous ^6^. However, this assumption has been challenged by two subsequent scientific findings. Firstly, the advent of phylogenetic analysis showed that these clades are evolutionarily more distant to each other than to other unsegmented taxa ^7, 8^. Secondly, the majority of segmented animals present a sequential mode of segmentation, where oscillations combined with embryo growth yield a rostral to caudal sequence of segments. Please note, however, that arthropods present alternative modes of segmentation, which are discussed elsewhere ^9– 11^.

**Figure 1.**
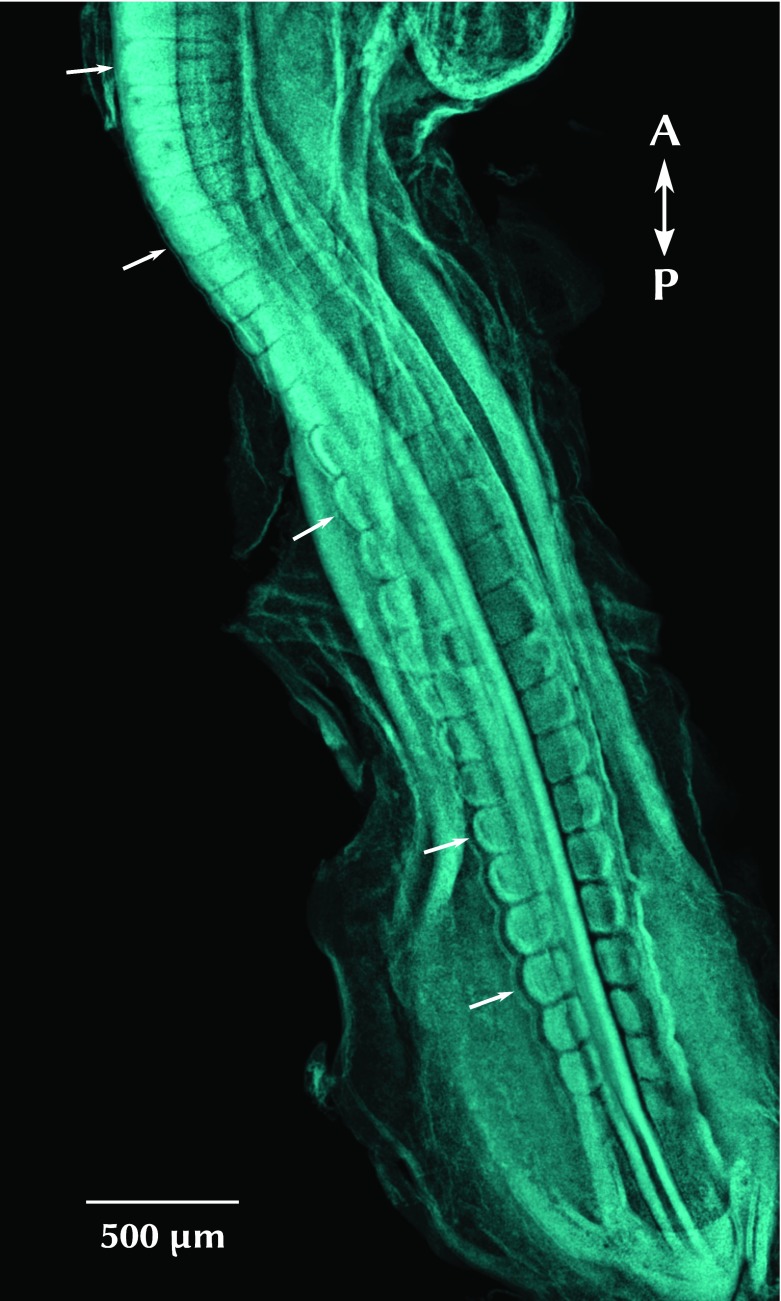
Chick embryo showing the segmented pattern along the anterior-posterior (A-P) axis. Embryo stage HH17, corresponding to 30 pairs of somites (age 52–64 hours). Dorsal view, stained with DAPI (cyan). Arrows indicate individual somites in different maturation stages.

These observations raise (at least) two intriguing questions: did the segmented body plan evolve once or multiple times, and how (and why) did the simultaneous segmentation evolve? Two alternative hypotheses have been postulated to address these questions (for reviews, see 6, 12– 14): (i) either sequential segmentation is an ancestral characteristic present in the last common ancestor of bilaterian animals, which remained conserved in the three segmented taxa and lost in all others ^15, 16^ (with the corollary that the simultaneous segmentation is derived from the sequential one) or (ii) segmentation arose separately in each of the three lineages and is therefore an example of convergent evolution. Different lines of evidence claim to corroborate each of these possibilities. For instance, some argue that the similar molecular pathways found in sequential segmentation of arthropods is indicative of homology ^17^; others suggest that these similarities may just be a consequence of the pleiotropy of signaling pathways such as Delta-Notch and thus misleading in this debate. Another interesting hypothesis is the possibility that these molecular similarities have been co-opted from a mechanism of posterior growth present in the common ancestor between the two groups ^18, 19^. This issue is very much still an open question. Accordingly, more than just asking how the different mechanisms of segmentation are related, one could ask: what are the main principles underlying them, what developmental and evolutionary changes lead to them, or even if there are other possible mechanisms we have not considered yet ^20^. These questions can be addressed by both broadening the diversity of organisms used to study segmentation ^21^ and exploring the potential of using computational evolution and synthetic biology ^22– 24^ to generate and test hypotheses (for an excellent example of a comprehensive theoretical study on the evolution of segmentation, see 16).

## The mechanism of segmentation

### The Clock and Wavefront model

In 1976, a theoretical model was proposed by Cooke and Zeeman to explain the dynamics of somitogenesis—the Clock and Wavefront model ^25^. This model proposes that cells of the PSM present an internal molecular oscillator—the clock—which is paired with a wavefront of cell differentiation progressing caudally as the embryo body axis elongates. This first conceptual model provided a possible explanation for how the temporal dynamics of a clock could be translated into a spatial pattern of somites.

### The molecular segmentation clock

Twenty years later, Palmeirim *et al*. ^26^ showed that *c-hairy1*, an avian homologue of the Drosophila *hairy* gene, was expressed dynamically in the PSM in a tissue-autonomous manner. Pulses of *c-hairy1* occur in individual PSM cells, with a 90-minute period. The fact that the individual PSM cells along the A-P axis are in different phases of the gene expression cycle (reviewed in 27) creates a kinematic wave (a wave in which transport is absent or negligible) of gene expression that sweeps rostrally, arresting in the anterior PSM, correlating in time and space with somite boundary formation ^26^. These results provided the first evidence supporting the long-held clock and wavefront hypothesis ^25^.

Nowadays, the clock is regarded as a genetic network composed of cell-autonomous and cell-to-cell signaling components, spanning multiple cells. Most genes involved are from the Notch, Fgf, and Wnt signaling pathways ^28, 29^ yet vary amongst model species such as chick, zebrafish, and mouse ^30^.

### Time to space translation

The segmentation clock oscillates in time with a sinusoidal curve, whose phase is locally synchronized between neighboring cells. According to the Cooke and Zeeman ^25^ model, as the wavefront passes down the PSM, it interacts with the clock, causing cells within the same period of oscillation to differentiate and become part of the same segment. This interaction is proposed to activate a developmental program that yields the formation of an epithelial somite in the anterior PSM.

**Figure 2.**
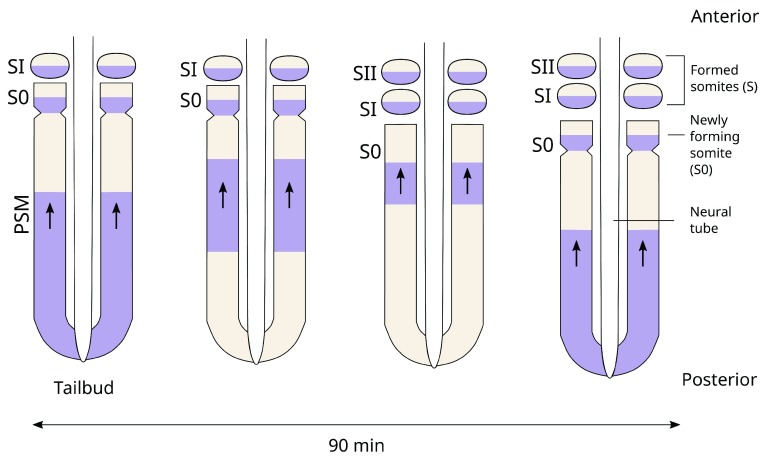
Time to space translation. Kinematic waves of gene expression sweep the A-P axis, arresting anteriorly, followed by somite formation.

This theoretical framework has provided an explanation for how the embryo could translate the information encoded in the temporal periodicity of oscillations of individual cells onto a spatially periodic pattern of segmentation from head to tail along the PSM. This model presents two important predictions: (i) each segment length ( *S*) is determined by the period of the clock ( *T*) and the velocity of the wavefront’s progress across the PSM ( *v*), i.e. *S* = *v T*, and (ii) the total number of segments in the embryo ( *n*) is set by the total duration of the segmentation process ( *d*) and the period of the clock ( *T*), i.e. *n* = *d/T* ^31^. This model places the period of the clock in a central role for determining the length and overall anatomy of the embryo ^25, 27, 32^. To form the correct number of somites, regardless of the differences in embryo size, both the period of the clock and the speed of the wavefront must be regulated in proportion to the overall length of the PSM ^25^.

Currently, new models contemplate the fact that the kinematic wave of *clock* expression narrows as it sweeps from the posterior to the anterior PSM, gradually slowing the clock anteriorly, with peaks of *clock* expression separated by one segment length in the anterior PSM ^27, 33– 36^.

### Alternative models for segmentation

Three relevant alternative mechanisms to transform oscillations into spatial stripes have been proposed:

(i) A Turing-Hopf mechanism has been shown by Hans Meinhardt to produce striped patterns from oscillations, provided that a gradient generates the first two stripes ^37^.
(ii) Similarly, Murray and co-workers demonstrated that an oscillator phase-gradient can also transform the oscillations into spatial patterns ^38^. This proposal gained more strength from a recent study suggesting that such a phase-gradient mechanism, rather than a wavefront, may be involved in freezing oscillations in vertebrate segmentation ^39^ ( *vide infra*).
(iii) More recently, Cotterell *et al.* ^40^ proposed the Progressive Oscillatory Reaction Diffusion (PORD) model. This elegant mathematical model is also a Turing mechanism, yet it overcomes the shortcomings of Meinhardt’s model ^37^; namely, the PORD model is able to describe the scaling of the somites with body size, and it can generate cell-autonomous oscillations without diffusion. Additionally, the PORD model displays enough flexibility to switch between simultaneous and sequential segmentation ^41– 43^, contrary to Murray’s coupled oscillators model ^38^.

Most notably, in all of these models, the wavefront is an emergent property of the time-space transformation rather than a causal agent ^16, 40^. Further experimental research is required to help define the models that provide the most insight into the segmentation mechanism.

## Recent experimental insights into vertebrate segmentation

### Segment scaling

Lauschke and colleagues proposed in 2013 a segment scaling mechanism based on a Phase-Gradient encoding, i.e. the gradual shift in the oscillation phase of PSM cells encodes information for the spatial patterning of the PSM tissue ^39^. Contrary to long-range molecular gradients (such as the wavefront proposed by Cooke and Zeeman), a phase-gradient describes the distribution of a dynamic cellular state. This provides a possible explanation for how the embryo maintains stable segment proportions despite overall changes in size, e.g. due to normal embryo growth or as observed when, after experimental reduction of embryo size, the total number of segments remains unaltered and the segments become proportionally smaller ^44, 45^.

Using their newly developed *ex vivo* experimental setting composed by a quasi-monolayer of mouse primary PSM cells (mPSM) combined with real-time gene expression imaging, the authors observed that (i) the period of oscillation in the central mPSM remains constant regardless of PSM length, (ii) the velocities of kinematic waves change linearly with overall mPSM length (larger samples display proportionally faster kinematic waves, indicating that oscillatory activity adapts to match the spatial context in which it occurs), and (iii) the phase-gradient slope is predictive of segment size.

Overall, this study suggests that segment size definition could be encoded at the level of phase differences between PSM cells, without the requirement for a molecular gradient, also known as a wavefront.

### Period of segmentation

In 2014, Soroldoni and co-workers set out to study the period of segmentation. Using real-time measurements of genetic oscillations in zebrafish embryos, they showed that the time scale of genetic oscillations is not sufficient to explain the period of segmentation as the segmentation clock postulates ^46^. Instead, the rate of tissue shortening provides the second time scale necessary to determine the period of segmentation through what they termed a “Doppler effect” modulated by a gradual change in the oscillation profile across the tissue.

Briefly, they found that (i) both anterior and posterior PSM oscillate, albeit with different amplitudes, (ii) oscillations in the posterior PSM are slower than in the anterior PSM (i.e. there is not a single well-defined period for the segmentation clock), (iii) the period of anterior PSM oscillations matches the period of segmentation, and (iv) there is a substantial shortening of the PSM over time, leading to a relative motion of the anterior end of the PSM toward the posterior end (where the waves arrest), hence creating a "Doppler effect" due to the shortening of the time interval between wave onset and arrest ^46^.

Overall, the authors conclude that the rhythm of sequential segmentation is an emergent property controlled by three variables: the period of the individual genetic oscillators, the change of the kinematic wavelength (i.e. oscillation profile), and the shortening of the PSM length. Notably, the authors remark that such a “Doppler effect” is incompatible with the previously proposed Phase-Gradient scaling mechanism, since the latter requires that the number of waves along the PSM remains constant, whereas this study shows that, at least in zebrafish, the number of waves changes between anterior and posterior PSM.

### Segmentation clock dynamics

In 2015, Shih *et al*. ^36^ studied, *in vivo*, the dynamics of the clock slowing down relative to somite boundary formation as it moves anteriorly through the PSM ^36^. For this, they followed the PSM segmentation clock oscillations in real-time, with single-cell resolution, in zebrafish embryos.

By focusing on cells that eventually form each somite boundary, they showed that (i) *in vivo*, the clock gradually slows down in the more anterior PSM cells, creating a distribution of oscillation phases—a spatial and temporal phase-gradient—where cells at one-somite distance are in opposite phases of clock expression, (ii) the clock wave increases in amplitude for the final two oscillations, and (iii) PSM cells oscillate until they incorporate into somites.

Most importantly, the authors discuss the lack of experimental evidence for the theoretical “arrest-front” causing oscillations to smoothly stop in the anterior PSM by the posteriorly progressing wavefront postulated by the Clock and Wavefront model ^25^. Instead, they propose the Clock Wave Stopping model. This proposal postulates that the segmentation clock itself may play a role in determining the wavefront by creating a signal directly encoded by the phase-gradient, with a two-somite periodicity ^36^. This is in line with the suggestion from Lauschke *et al*. ^39^.

This model is based on one important observation: within a given forming somite, individual cells stop oscillating in discrete groups in a posterior to anterior direction (P-A), i.e. in the same direction as the waves of *clock* expression and not in the opposite direction, as would be expected from an A-P wavefront stopping signal. In this Clock Wave Stopping model, cells in the anterior PSM continue to oscillate with their neighbors, regardless of future somite position, consistent with the observations that synchrony is regulated by Delta-Notch interactions ^47, 48^.

### Autonomous segmentation clock

In early 2016, Webb *et al*. ^31^ addressed the longstanding question of whether individual PSM cells are able to sustain autonomous oscillations without the external input from neighboring cells.

To this end, the authors recorded the expression of *Her1* in single cells from a transgenic reporter cell line obtained from the tailbud of zebrafish, showing that the individual cells are capable of autonomous genetic oscillations *in vitro*, in the absence of intercellular communication, albeit with substantial variability (noise) ^31^. When comparing these autonomous oscillator data with the oscillations at the tissue level in intact zebrafish embryos (from Soroldoni *et al*. ^46^), the authors concluded that individual cells have a longer period (ca. twofold slower) and are less precise than the population at the tissue level. This shows the importance of synchronization and coupling for the proper working of the segmentation clock.

### Self-organized spatiotemporal waves

Also in 2016, Tsiairis and Aulehla ^49^ presented compelling evidence that PSM cells can self-organize from disordered initial conditions. For this study, the authors dissociated the PSM from several mouse embryos into single cells and used the randomized cell suspension to generate dense cell re-aggregates. These were then cultured and subjected to real-time imaging and quantification of signaling activity using the dynamic Notch signaling reporter LuVeLu (Venus fluorescence driven from the lunatic fringe promoter) ^49^.

They reported several relevant findings, from which we highlight the following: (i) after 5–6 hours of culture, cells synchronize and exhibit in-phase oscillations in multiple foci that formed within each re-aggregate, (ii) self-organized foci recapitulate spatiotemporal organization of *in vivo* PSM, indicating that each focus represents miniature PSM (emerging PSM) that forms spontaneously upon randomization and re-aggregation, (iii) the collective synchronization of the cells corresponds to the arithmetic average of the input oscillator frequencies (i.e. it depends on frequencies of the input cell population), as predicted in models for coupled phase oscillators ^50^, rather than matching the highest frequency of potential pacemaker cells, and (iv) upon DAPT treatment (a chemical inhibitor of Notch signaling), randomized PSM cells fail to synchronize, in agreement with previous *in vivo* findings ^51^; however, the individual cells maintained oscillatory activities with amplitudes similar to the untreated ones.

Overall, this report indicates that, after randomization, individual mouse PSM cells self-organize into ordered macroscopic miniature PSM structures that are capable of tuning oscillation dynamics in response to surrounding cells, leading to collective synchronization with an average frequency, re-establishing wave-like patterns of gene activity.

### Control of the segmentation clock period

Later in the same year, Liao *et al*. ^52^ asked whether an increase in Delta-Notch signaling causes a change in the segmentation period. To answer this, they generated transgenic zebrafish lines with a range of extra copies of a transgene containing the *deltaD* locus (together with its full genomic regulatory region) and measured the segmentation period by multiple embryo time-lapse microscopy ^52^.

They found that only the highest level of DeltaD expression (in the Damascus zebrafish line containing ca. 100 copies of deltaD-venus transgene) produces altered oscillating gene expression wave patterns and shorter segmentation periods (6.5% faster), generating embryos with more, and shorter, body segments. Moreover, the effect on period was lost by incubating Damascus embryos with DAPT (Notch intracellular domain blocker), showing that the observed phenotype is indeed modulated by the overexpression of Delta-Notch molecular players.

The contribution of the proposed "Doppler effect" ^46^ on the observed change in segmentation period was also evaluated, showing that the alteration of the wave pattern, and not changes in the rate of tissue shortening, is responsible for the majority of the observed period difference.

Finally, the authors put forward a simplified model of coupled oscillators with time delay to explain the shorter segmentation period. Briefly, the coupling strength (i.e. the number of activated ligand–receptor pairs over time) is expected to increase as a consequence of the over-expression of DeltaD, and the time delay in the coupling is not negligible (i.e., it is significant the time taken to transfer the signal from one cell to another owing to the time required to synthesize and traffic Delta proteins). The numerical simulation of such parameter changes leads to a stable time-periodic wave pattern with an increased number of waves and a shortened anterior wavelength, as experimentally observed in the Damascus line.

Thus, this study hints that the Delta-Notch signaling, besides synchronizing oscillators, seems to alter the segmentation period by affecting the wavelength of the tissue’s spatial pattern. However, this issue is not easy to address, since several alternative outcomes arise from changing the Delta-Notch signaling. For instance, a lengthening of the period due to a reduction in Delta-Notch signaling is observed in zebrafish. However, mouse segmentation is faster with the blockade of Notch, an outcome predicted only if the length of the time delay in the coupling was shorter than half the period ^34, 53^.

## Open questions

Segmentation has been extensively studied over the years. However, some fundamental questions remain unanswered, while new ones are being asked, most of them fueled by newly developed experimental techniques such as high-resolution live imaging, single cell tracking systems, and customized *ex vivo* cell culture assays. Here we compile some of the open questions regarding the inner workings of the vertebrate segmentation clock. The questions are grouped according to Oates’ Three Tier model, which organizes the components of the segmentation clock into three different scales: single cell oscillators, local synchronization, and global control of timing and pattern ^27^.

### Cellular oscillator

**Q1** | Recently, it has been shown in zebrafish that single cells carrying a *her1-yfp* transgene can oscillate autonomously ^31^. However, it remains unknown if this is a particular feature of this organism, or if it is an intrinsic characteristic of the segmentation clock cells and hence observed in other species (this has been attempted in chick ^54^ and mouse ^55^ but without definite conclusions owing to the technological limitations at the time).

### Local synchrony

**Q2** | Wave patterns arising from individual oscillators require the establishment of a spatial profile in their phases. How are these wave patterns regulated?

**Q3** | The Delta-Notch intercellular signaling pathway is associated with multiple processes, namely lateral inhibition, border formation, and local synchronization. How is the Delta-Notch signaling accomplishing these multiple outcomes?

### Global control

**Q4** | In vertebrates, the wavefront has been shown to be influenced by gradients of Wnt and Fgf signaling coming from the caudal end and a counter-gradient of retinoic acid (RA) from the somites. How do oscillating cells use and interpret these signaling gradients as stop signals to arrest the oscillation?

**Q5** | In vertebrates, embryo segmentation occurs simultaneously with body elongation. Is it possible to disentangle these two processes in order to study them separately?

**Q6** | The first five somites are not sequentially formed like the ones from the trunk ^43^. Instead, they form almost simultaneously and without cyclical expression of "clock genes" ^56^. Is there an alternative mechanism responsible for occipital somite formation?

**Q7** | Recently, relevant challenges have been made to the clock and wavefront model. Particularly important is the fact that, in order to fully grasp the global mechanism underlying segmentation, it is necessary not only to study gene expression dynamics but also to integrate cellular processes like division, movement, and differentiation ^57^. Such a comprehensive theoretical framework would generate an invaluable tool to create new hypotheses and test old ones.
